# Pattern of Pediatric Ocular Trauma in Kashan

**DOI:** 10.5812/atr.5302

**Published:** 2012-06-01

**Authors:** Davood Aghadoost, Mohammad Reza Fazel, Hamid Reza Aghadoost

**Affiliations:** 1 Trauma Research Centre, Kashan University of Medical Sciences, Kashan, IR Iran; 2 Department of Ophthalmology, Kashan University of Medical Sciences, Matini Hospital, Kashan, IR Iran; 3 Department of surgery, Shahid Beheshti University of Medical Sciences, Tehran, IR Iran

**Keywords:** Hospitalization, Injury, Pediatric, Patients

## Abstract

**Background:**

Ocular trauma is a significant health problem in pediatric patients.

**Objectives:**

The aim of this study was to analyze the characteristics of ocular-trauma-related hospitalization of children in Kashan.

**Patients and Methods:**

This descriptive, cross-sectional study included 131 children aged less than 16 years with ocular trauma, who were admitted to the Matini Hospital at the Kashan University of Medical Sciences between April 2006 and March 2009. After admission, detailed ocular examination was performed, and their ocular trauma was classified according to the International Ocular Trauma Classification and Birmingham Eye Trauma Terminology systems.

**Results:**

Mean age of the patients was 7.8 ± 2.2 years (age range, 0–16 years), and male to female ratio 5:1. The most common cause of admission was hyphema (38.1%), followed by corneoscleral laceration (27.5%). Ocular trauma most commonly occurred at home (43%), and 69% of the patients presented to the emergency room within 24 h of injury. In 30% of the patients, initial visual acuity at the time of presentation was less than 20/200 (Figure 1).

**Conclusions:**

Ocular trauma is a major cause of unilateral blindness, especially in young boys, and hence, preventive measures and education is required.

## 1. Background

Eye injuries are significant health problem leading to morbidity and blindness, especially in children (1). Thirty-five percent of all cases of ocular trauma occur in children under the age of 17 years (2). The frequency of hospitalization due to ocular trauma differs between developed and under-developed countries; for example, it is 8 per 100,000 people in Scotland and 33 per 100,000 in Guiana (3-9). In children, ocular trauma is the most common cause of decreased vision in one eye or noncongenital blindness. In children under the age of 3 years, the most common cause of enucleation is ocular trauma (2, 10, 11). Ocular trauma in children is different from that in adults. Children with ocular trauma usually have no visual complaints and gradually develop amblyopia (10, 11). 

## 2. Objectives

In various studies, many aspects of ocular trauma have been evaluated. We aimed to identify better means to plan and implement strategies for eye care and safety for preventing eye injuries by determining the patterns of ocular trauma that lead to hospitalization of children in Kashan.

## 3. Patients and Methods

In this descriptive, cross-sectional study, we included 131 patients aged less than 16 years, who had been admitted to the Matini Hospital at the Kashan University of Medical Sciences for the management of eye injuries. The necessity for admission to the hospital was determined by an ophthalmologist after detailed eye examinations in the emergency room (ER). We determined the initial visual acuity in all patients, examined the globe and its adnexa, and performed slit-lamp examination and dilated fundus examination (if possible). In very young and uncooperative children, visual acuity was tested with age-appropriate methods. We also recorded characteristics such as age, sex, interval between trauma and presentation to the ER, type of activity at the time of injury, the type and extent of ocular trauma, whether the patient was managed with or without surgical intervention, and the ocular condition (visual and anatomical status) at the time of discharge. The types and sites of ocular trauma are listed in *Table 1*. The classification of ocular trauma was based on modified form of International Ocular Trauma Classification and Birmingham Eye Trauma Terminology (12, 13).

## 4. Results

The study population comprised 131 patients with a mean age of 7.8 ± 2.2 years (range, 0–16 years) who were admitted to the ophthalmology ward for ocular trauma. Seventy percent of the patients were in the age group of 6–15 years, and 82.4% were boys. Characteristics of the patients have been listed in *Table 2*. 

**Table 1.** Type and Mechanism of Injuries in 131 Children With Ocular Trauma Hospitalized at the Matini Hospital in Kashan

	No. (%)
Lidaud canalicular laceration	14 (10.7)
Corneal laceration	23 (17.6)
(anterior segment)	
Corneascleral laceration (anterior-posterior segment)	36 (27.5)
Intraocular foreign bodies (penetration)	8 (6.1)
Hyphema (blunt)	50 (38.1)
Total	131 (100)

**Figure 1.**
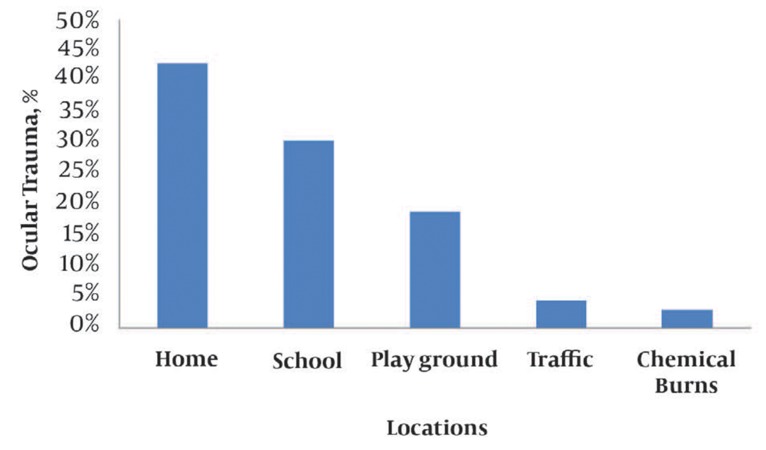
Location of occurrence of ocular trauma

The sites of ocular injuries are shown in the bar graph (*Figure 1*). In most cases, the injuries occurred at home. In 3% of the patients, the cause of ocular damage was chemical burns.

All the patients were followed up at an out-patient clinic for at least 6 months, and their final visual acuity was recorded.

The duration of hospital stay was less than 3 days in 73% of the patients and less than 7 days in all the patients. Sixty four percent of the patients required surgery (one or more sessions). The best corrected visual acuity of the patients, 3 months after trauma has been presented in *Table 3*. 

**Table 2.** Characteristics of the Children With Ocular Injuries Admitted to the Matini Hospital in Kashan During 2006–2009

	No. (%)
Age group, y		
0–2	10 (7.6)
3–5	31 (23.7)
6–9	30 (23)
10–12	28 (21.4)
13–15	32 (24.3)
Age range, y	
0–16	131 (100)
Interval between trauma and presentation to ER^a^	
≤ 24, h	90 (68.7)
24–48, h	33 (25.2)
≥ 48, h	8 (6.1)
Initial visual acuity during presentation to ER^a^	
≥ 20/40	25 (19)
20/30-20/200	42 (32.1)
≤ 20/200	64 (48.9)

^a^Abbreviation: ER, Emergency Room

**Table 3.** Initial and Final Visual Acuities of Patients

Visual acuity	Initial Visual Acuity (During Presentation to ER ^a^), No. (%)	Final Visual Acuity (at Discharge), No. (%)	3 Months After Trauma (OPD a), No. (%)
≥ 20/40	25 (19)	63 (48)	72 (55)
20/30-20/200	42 (32.1)	31 (23.6)	34 (26)
≤20/200	64 (48.9)	37 (28.3)	25 (19)
Total	131 (100)	131 (100)	131 (100)

^a^Abbreviations: ER, Emergency Room; OPD, Out patient department

## 5. Discussion

In this study, pediatric ocular trauma was fivefold more common in boys, as has been noted in other studies throughout the world (11, 14-16). Boys were usually more susceptible to ocular damage because of the nature of their activities and presumably less supervision by their families. The major type of injury necessitating hospitalization was hyphema due to blunt trauma, accounting for 38% of all the cases. In a large study on pediatric ocular trauma in the USA by Brophy *et al.* in 2006, the major type of ocular injury leading to hospitalization was hyphema (2). In another study by Carolina M *et al*., 60% of the children hospitalized for ocular trauma had hyphema (17). Penetrating and ocular trauma were the second most common cause of admission in their study, as in other researches (2, 11, 14, 15).

The present study showed that ocular injury occurred most commonly at home (43%), followed by school and playground; this is consistent with the observations in other studies (2, 14). In another study conducted in Yazd, a central province in Iran, by Shoja *et al.* (11), ocular trauma occurred most commonly at home; most of the other incidences occurred in traffic accidents. In our study, traffic-related ocular injury accounted for only 4.5% of all the cases. In another similar study (18, 19) by Chin-Hsing *et al.* in Taiwan, the most frequent causes of ocular trauma in pediatric patients were falls, followed by assaults and chemical burns (14).

In the present study, 67% of the children with ocular trauma presented to the ER within 24 h of undergoing trauma, and 94% within 48 h, a trend similar to that observed in studies by McCarthy in USA (8) and Dandrang in India (5). In another study by Tarique *et al*., 67.3% of the patients with ocular trauma were referred to a hospital in the first week after the trauma because of logistic and socioeconomic reasons (18). In another study by Saxena in India, 24% of the patients had presented 6 h after the injury, and 34% after more than 24 h after injury (20).

The initial visual acuity of the damaged eyes of patients (48%) at the time of presentation to the ER was less than 20/200, which is similar to the observation from other studies in Iran, Singapore and USA (2, 4, 7, 11). The final visual acuity was ≥ 20/40 in 55% of cases, which is again similar to the results from other studies (5, 11). Appropriate management of ocular trauma improves the final visual outcome and anatomy of the damaged eyes and adnexa. Ocular trauma is one of the most important avoidable causes of decreased vision and blindness leading to lifelong morbidity. Hence, there is a need to develop prevention measures with high supervision of patients at home and by instructors at preschools and schools. After occurrence of injuries, prompt reference and presentation to ER and appropriate management are very effective in restoring the vision and anatomy of the eye and its adnexa.
